# Comparative Anti-Cancer and Anti-Inflammatory Activities of Essential Oils from the Bark and Flower of *Magnolia officinalis* Rehd. et Wils

**DOI:** 10.3390/foods13132074

**Published:** 2024-06-29

**Authors:** Ke-Xin Hao, Yun-Fang Hao, Jie Zhang, Xi-Lin Xu, Jian-Guo Jiang

**Affiliations:** 1 College of Food and Bioengineering, South China University of Technology, Guangzhou 510640, Chinaxuxilin@scut.edu.cn (X.-L.X.); 2 Jiangmen Key Laboratory of Traditional Chinese Medicine Ingredients and Their Mechanisms of Action, Guangdong Jiangmen Chinese Medicine College, Jiangmen 529000, China

**Keywords:** essential oils, *Magnolia officinalis* Rehd. et Wils, anti-inflammation, anti-cancer, extraction

## Abstract

This study was designed to compare the antioxidant, antitumor and anti-inflammatory effects of essential oils from the bark and flower of *Magnolia officinalis* Rehd. et Wils. Distillation extraction and steam distillation were used to extract EOs from the bark and flower. The results showed that the contents of EOs of SDE-F and SDE-B were much higher than that of SD-F and SD-B. EOs from the bark were rich in eudesmol (especially α-eudesmol) and exhibited a stronger antioxidant effect than the flower. The anti-tumor effects of SD-B and SD-F on HepG2 and MDA-MB-231 cells were better than that of SDE-B and SDE-F. The inhibitory rates of SD-B and SD-F on MDA-MB-231 cells were 59.21% and 48.27%, exceeding that of positive control 5-fluorouracil (47.04%) at 50 μg/mL. All four EOs exhibited excellent anti-inflammatory activities through the regulation of nitric oxide production and pro-inflammation cytokines in LPS-induced RAW 264.7 cells and they also remarkably suppressed the mRNA expressions of nitric oxide synthase, IL-6 and TNF-α at the concentration higher than that of positive control dexamethasone. These results indicated significant differences in the composition, and anti-inflammatory and anti-tumor activities of EOs extracted by different methods and provided a theoretical basis for their development and utilization.

## 1. Introduction

*Magnolia officinalis* Rehd. et Wils, belonging to Magnoliaceae, has been used as an edible and medicinal material for thousands of years in China and Japan [1]. The stem bark of *M. officinalis*, commonly called Houpu, was usually used for the treatment of bronchial asthma, anxiety and gastrointestinal symptoms including acute diarrhea, cramping abdominal pain, regurgitation, vomiting and dyspepsia [2,3]. Moreover, the extracts from *M. officinalis* possess a variety of pharmacological activities such as anti-inflammatory [4,5] and anti-cancer [6] activities, and lipase inhibition [7]. The flower of *M. officinalis* also exhibited excellent antioxidant effects [8].

So far, most of the studies involving *M. officinalis* focused on the polyphenols such as magnolol and honokiol, which are the main active substances with very high content in *M. officinalis* [9,10]. However, both the stem bark and flower of *M. officinalis* also have plenty of essential oils (EOs), whose bioactivities have been less studied.

Accumulating studies have confirmed that EOs exhibit many pharmacological properties including anti-anxiety, anti-depressant, antioxidant, antibacterial, antifungal and, especially, anti-inflammatory activities [11,12,13,14,15]. Several methods were used to extract EOs from plants such as steam distillation (SD), simultaneous distillation extraction (SDE), heating reflux extraction (HRE) and supercritical fluid extraction (SFE) [16,17,18,19]. Among them, the SD method was a classical extraction method recorded in the China Pharmacopoeia, and SDE was one mature and conventional method, owing to its simple operation and low cost. Although SFE has the advantages of low extraction temperature and high extraction efficiency, it required a high equipment investment and operating cost [20].

In this study, the SD and SDE methods were used to extract EOs from the *M. officinalis* bark and flower, and Gas Chromatography–Mass Spectrometry (GC–MS) was used to analyze the EO components. Subsequently, the anti-oxidant, anti-inflammatory and anti-cancer effects of EOs were estimated using relevant assays. The aim of the present study was to extract and analyze the essential oils of the flowers and bark of *M. officinalis* to further evaluate the pharmacological effects by comparison of its activities such as anti-tumor and anti-inflammatory activities, and to provide some insights for the evaluation of *M. officinalis*.

## 2. Materials and Methods

### 2.1. Plant Materials

The flower and bark of *M. officinalis* were purchased from the Qingping traditional Chinese medicine market in Guangzhou and were authenticated by the South China Botanical Garden, Chinese Academy of Sciences, where voucher specimens (voucher specimen number 60638) were kept. After being dried at 60 °C in a hot-air oven, the plant materials were ground to a fine powder using a lab mill. Prior to dry preservation, the powder was passed through a 60-mesh screen.

### 2.2. Extraction of Essential Oil

Two extraction methods, SD and SDE, were used for the extraction of EOs from *M. officinalis*. Extraction was performed in triplicate.

#### 2.2.1. SD Method

Firstly, the dried powder of the *M. officinalis* bark and flower (400 g) was soaked in distilled water (4000 mL) (solid/liquid (*w/v*) = 1:10) for 1 h and extracted by steam distillation for another 6 h. Secondly, the separate layer was extracted by an equal volume of diethyl ether after natural cooling for 30 min. Thirdly, anhydrous sodium sulphate was used to remove water. The EOs extracted from *M. officinalis* bark and flower were regarded as SD-B and SD-F, respectively, and then kept in brown glass bottles at 4 °C until the moment of analysis.

#### 2.2.2. SDE Method

Dried materials (300 g) were placed into a 5 L round-bottom flask with 3 L distilled water and extracted by steam distillation at 100 °C for 6 h. Simultaneously, 60 mL dichloromethane was placed into the solvent flask under a constant-temperature water bath at 50 ℃ using a modified Likens–Nickerson apparatus. After natural cooling for 30 min, the extracted liquor was collected and dried over anhydrous sodium sulfate, which was subsequently filtered out. Then, a rotary flash evaporator was used to remove the dichloromethane. The EOs extracted from the *M. officinalis* bark and flower were obtained as SDE-B and SDE-F, respectively.

### 2.3. GC–MS Analysis

The EOs from *M. officinalis* were analyzed by GC–MS based on the published protocol with minor modifications [21]. Briefly, four EO samples (10 μL) were diluted to 500 times by dichloromethane; then, anhydrous sodium sulfate was added over night. Prior to GC–MS being used for sampling analysis, all samples were filtered through a membrane. Chromatographic conditions were as follow: HP-5MS column (30 m × 0.25 mm × 0.25 mm); column temperature varied from 50 °C (held for 8 min) to 120 °C at 10 °C/min, and was then raised to 200 °C at 5 °C/min, and then increased to 280 °C (held for 10 min) at 15 °C/min; injector temperature of 280 °C with an injection volume of 1 μL; split ratio of 5:1; pressure of 12.051 psipis; Mass spectrometer conditions were as follows: electron impact ion source; ion source temperature of 230 °C; quadrupole temperature of 150 °C; solvent delay of 3.50 min; electron energy of 70 eV; mass scan range of *m/z* 35–550.

### 2.4. Antioxidant Activity

#### 2.4.1. Reducing Power assay

The production of Perl’s Prussian blue-colored complex was used as an indicator of the reducing power [22]. Initially, 1 mL of EO samples at various concentrations of 6.25–200 μg/mL was mixed with 2.5 mL phosphate buffer (0.2 M, pH 6.6) and 2.5 mL of potassium ferricyanide solution (1%, *w/v*); then, the mixtures were reacted at 50 °C for 20 min. After cooling in the ice bath, 2.5 mL of trichloroacetic acid (10%, *w/v*) solution was added to stop the reaction immediately. Subsequently, the mixtures were centrifugated at 3000 rpm for 10 min; then, 2.5 mL of the supernatant was mixed with 2.5 mL of distilled water and 0.5 mL of ferric chloride solution (0.1%, *w/v*). After mixing and standing for 10 min, the mixtures were measured spectrophotometrically at 700 nm.

#### 2.4.2. DPPH• Radical Scavenging Activity Assay

Assays for 1,1-diphenyl-2-picrylhydrazyl radical (DPPH•) radical scavenging were determined by the method of Villaño et al. [23]. Briefly, 20 μL of EO samples at concentrations of 6.25–200 μg/mL and 180 μL DPPH solution (150 μmol/L) were added into a 96-well plate. After that, the reaction mixtures were incubated for 20 min at 37 °C. The absorbance at 517 nm was measured. Several groups were set as follows: blank control group (absolute ethanol plus DPPH solution), positive control group (V_C_ plus DPPH solution) and background control group (absolute ethanol plus sample).

DPPH• scavenging rate (%) = [1− (OD_sample_ − OD_background control_)/OD_blank control_] (1)

#### 2.4.3. ABTS•+ Radical Scavenging Activity Assay

Assays for 2,2′-azinobis(3-ethylbenzothiazoline-6-sulfonic acid) (ABTS•+) radical scavenging were determined by a previous method with slight modifications [24]. The ABTS mother liquor was prepared by mixing 4.9 mmol/L potassium persulfate solution with 7 mmol/L ABTS solution (1:1; *v/v*). Then, 20 μL of different EO sample concentrations were mixed with 180 μL of ABTS working liquor (0.25 mmol/L); the obtained solution was incubated at 30 °C for 6 min. Eventually, the absorbance was taken at 734 nm. The blank control group (distilled water plus ABTS working liquor), positive control group (V_C_ plus ABTS working liquor) and background control group (distilled water plus sample) were set. The scavenging percentage of ABTS•+ was calculated on the basis of the following equation:

ABTS•+ scavenging rate (%) = [1− (OD_sample_ − OD_background control_)/OD_blank control_] (2)

#### 2.4.4. FRAP Assay

Assays for the ferric reducing antioxidant power assay (FRAP) were measured using the method of Benzie and Strain [25]. The 1,3,5- tri(2-pyridyl)-2,4,6-triazine (TPTZ) working solution (0.83 mmol/L) was prepared by mixing TPTZ solution (10 mmol/L), ferric chloride solution (20 mmol/L) and sodium acetate solution (0.3 mol/L) (1:10:1; *v*/*v*) when they were to be used. Similar to the above methods, 20 μL of different EO sample concentrations or FeSO_4_ solution were mixed with 180 μL of the TPTZ working solution. After incubation at 37 °C for 10 min, the absorbance of the reaction mixture was detected at 593 nm. The blank control group (distilled water plus TPTZ working solution) and positive control group (V_C_ plus TPTZ working solution) were set. The FRAP value of EOs samples were calculated according to the FeSO_4_ standard curve.

#### 2.4.5. •OH Scavenging Activity Assay

Hydroxyl radical (•OH) scavenging activity was determined by a previous method with some modifications [26]. Initially, 1 mL of the tested samples was mixed with 1 mL of H_2_O_2_ (60 mmol/L) and 1 mL of FeSO_4_ (9 mmol/L); then, 3 mL of salicylic acid-ethanol (9 mmol/L) was added. After that, the mixtures were incubated for 15 min at 37 °C. Finally, the absorbance at 510 nm was detected, and •OH scavenging activity was expressed as follows:

•OH scavenging rate (%) = [1− (OD_sample_ − OD_background control_)/OD_blank control_] (3)

where OD_blank control_ was the absorbance of the control (distilled water), OD_background control_ was the absorbance of the background control (sample, FeSO_4,_ salicylic acid-ethanol and distilled water) and OD_sample_ was the absorbance in the presence of different samples.

### 2.5. Cell Culture

Human hepatocyte cells L02, human hepatocarcinoma cells HepG2, human lung carcinoma cells H1299, human breast cancer cells MDA-MB-231 and murine macrophages RAW264.7 were maintained in DMEM supplemented with 10% (*v/v*) FBS and antibiotics (100 IU/mL penicillin and 100 μg/mL streptomycin) at 37 °C in a humidified atmosphere containing 5% CO_2_. All EO samples were dissolved in DMSO and filtered through a 0.25 μm filter membrane. During the experiment, the samples were diluted in DMEM to various working concentrations.

### 2.6. MTT Assay

The cell viability was measured by MTT colorimetric assay. All EO samples were dissolved in DMSO at the concentration of 10 mg/mL and diluted by DMEM to various working concentrations. Cells with a density of 3 × 10^3^ cells per well were cultured in 96-well plates with or without EO samples (1.5625, 3.125, 6.25, 12.5, 25, 50 μg/mL). After 24 h, the culture medium was discarded, and 200 μL of MTT (dissolved in PBS at 5 mg/mL) in DMEM was added. A total of 4 h later, the supernatant was discarded and 150 μL of DMSO was added. Finally, OD_490_ values were measured on a microplate reader to evaluate the cell viability. The traditional anti-cancer drug 5-Fu was used as a positive control, and the concentrations were the same as for the EO samples.

### 2.7. Determination of NO, IL-6 and TNF-α by ELISA in RAW264.7 Cells

Briefly, RAW264.7 cells were plated in 96-well plates (5 × 10^4^ cells/well) for 24 h for adherence; then, lipopolysaccharide (LPS) at a final concentration of 1 μg/mL and EO samples at different concentrations including 12.5, 25 and 50 μg/mL were added, besides the control. After being incubated for 24 h, the supernatants were collected. The production of NO and the secretion of IL-6 and TNF-a were determined by the commercially available ELISA kits according to the previous method [27]. Dexamethasone (DXM) (50 μg/mL) was used as a positive control.

### 2.8. RT-qPCR Analysis

After incubation for 12 h with EO samples and LPS, total RNA was extracted by the Trizol reagent. The RNA was reverse transcribed using the Revert Aid First Strand Synthesis Kit and amplified using the FastStart Universal SYRB Green qPCR Kit conducted by ABI real-time fluorescent quantitative PCR. The RT-qPCR primers were listed as follows: inducible nitric oxide synthase (iNOS) (forward, 5′-CGGCAAACATGACTTCAGGC-3′; reverse, 5′-GCACATCAAAGCGGCCATAG-3′), IL-6 (forward, 5′-TACTCGGCAAACCTAGTGCG-3′; reverse, 5′-GTGTCCCAACATTCATATTGTCAGT-3′) and TNF-α (forward, 5′-GGGGATTATGGCTCAGGGTC-3′; reverse, 5′-CGAGGCTCCAGTGAATTCGG-3′). The relative quantification study method was used for data analysis after completion of the experiment to obtain the relative expression of the target genes.

### 2.9. Statistical Analysis

All experiments were performed in triplicate. Results shown in this article are expressed as mean ± SD. The significances of differences between groups were evaluated by analysis of variance (ANOVA). Among them, *p* < 0.05 (*) indicated a statistically significant difference, and *p* < 0.01 (**) was considered to be a highly significant difference.

## 3. Results and Discussion

### 3.1. Comparative Analysis of Essential Oil Compounds

SDE-F and SDE-B extracted by the SDE method from the *M. officinalis* flower and bark were yellowish with a pleasant odor, and the yields were 0.121 ± 0.0012% and 0.223 ± 0.0017%, respectively, based on the dried sample powder. Meanwhile, the yields of SD-F and SD-B extracted by the SD method were 0.110 ± 0.0015% and 0.218 ± 0.0012% from the *M. officinalis* flower and bark, which were lower than that of SDE-F and SDE-B. Consistent with the results of the yields, the component numbers of SDE-F (29) and SDE-B (26) were also much higher than SD-F (24) and SD-B (19) (Table 1). These results indicated that the SDE method showed a higher yield and more components on the extraction of Eos compare to the SD method, which was consistent with a previous study [28]. In addition, the EO from the *M. officinalis* flower also contained more components than that of bark.

**Figure 1:**
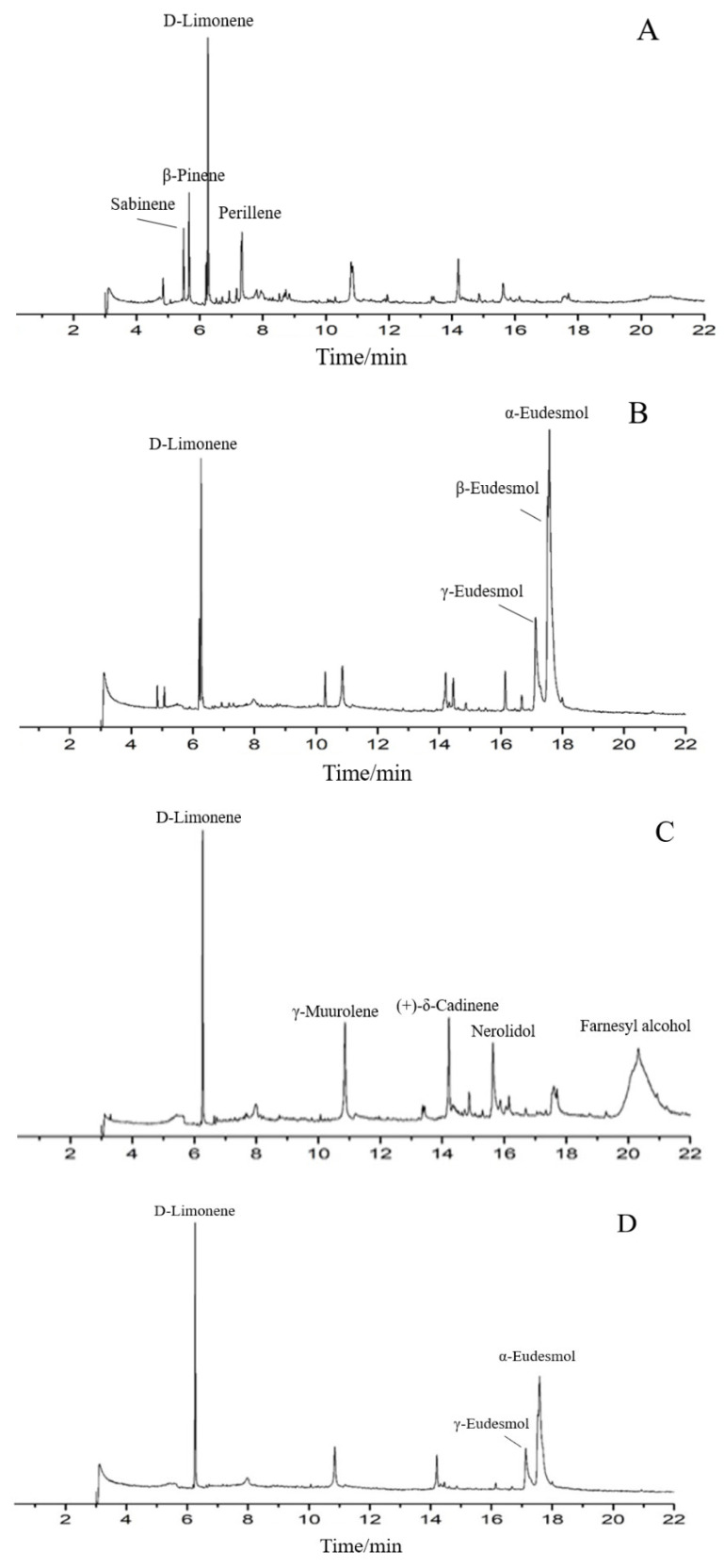
Total ion chromatograms of GC/MS of essential oils from t *Magnolia officinalis* Rehd. et Wils. ((**A**) SDE-F; (**B**) SDE-B; (**C**) SD-F; (**D**) SD-B).

The phytochemical constituents of four EO samples from *M. officinalis* are presented in Table 1 and Figure 1. The prominent components of SDE-F were characterized as D-Limonene (31.17%), β-Pinene (12.25%), Perillene (8.32%) and Sabinene (7.91%), respectively. Farnesyl alcohol and D-Limonene, together representing 57.33%, were two main compounds in SD-F. On the other hand, the main abundant compounds of SDE-B were α-Eudesmol (40.17%), β-Eudesmol (12.55%), γ- Eudesmol (12.39%) and D-Limonene (11.37%). α-Eudesmol was also the main compound, representing 40.36% of the total EOs in SD-B, followed by D-Limonene (21.35%) and γ- Eudesmol (12.9%). In general, D-Limonene, α-Eudesmol and β-Eudesmol were the main components in EOs from *M. officinalis*. In addition, four EOs from *M. officinalis* all contained D-Limonene, β-Pinene, α-Eudesmol and β-Eudesmol.

As is shown in Table 1, approximately more than ten components including D-Limonene, β-Pinene, Caryophyllene oxide, Linalool et al. were detected from the *M. officinalis* flower by two extraction methods; among them, D-limonene occupied the largest proportion, indicating that D-limonene could be considered as one of the main components of EOs from the *M. officinalis* flower. On the other hand, Eudesmol (especially α-Eudesmol) was the main component in SDE-B and SD-B extracted from *M. officinalis* bark, as well as D-limonene.

### 3.2. Antioxidant Activity

The overproduction of oxidized substances could result in oxidative stress in a biological system, followed by an overproduction of pro-inflammatory cytokines, causing an increased risk of several illnesses in humans. Thus, a growing amount of research has been conducted to explore anti-oxidant activities, and natural products were widely recognized as excellent antioxidants owing to their few side effects. For example, a previous study reported that the scavenging activity of the EO of clove buds on DPPH radicals was in the range of 15.4–60.4% at the concentration of 100–1000 µg/mL [29]. Resveratrol, a famous antioxidant, has been found in various plants such as berries, grapes, peanuts, pistachios and plums [30,31].

**Figure 2:**
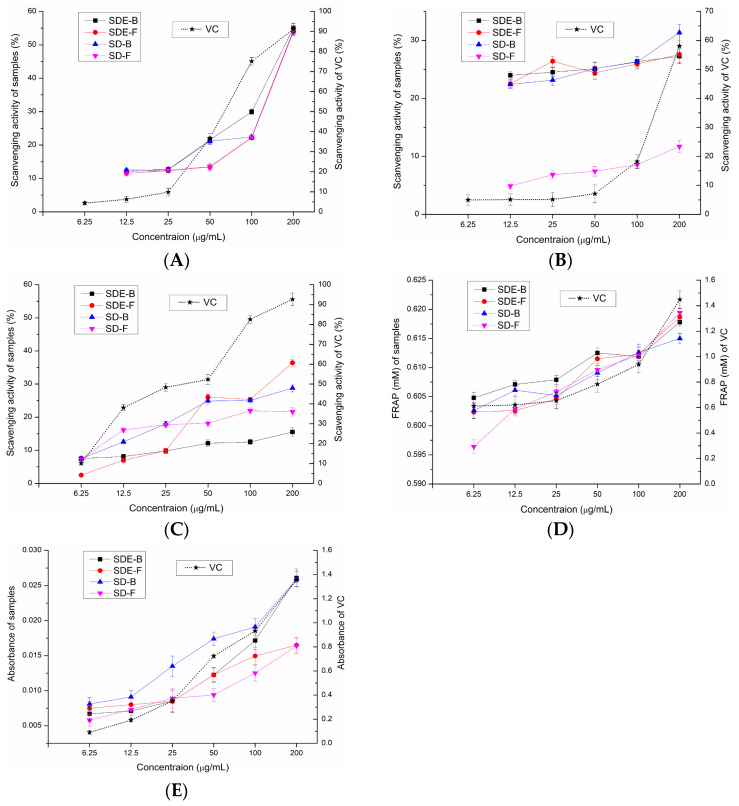
Scavenging effects of SDE-B, SDE-F, SD-B and SD-F on (**A**) DPPH•, (**B**) ABTS•+ and (**C**) •OH and their (**D**) FRAP value and (**E**) total reducing power.

In this study, the antioxidant activities of EOs from the *M. officinalis* flower and bark by the SD and SDE methods have been evaluated in vitro. Samples of SD-F and SD-B extracted by the SD method and SDE-F and SDE-B extracted by the SDE method were evaluated for their antioxidant activities using total reducing power, DPPH• radical scavenging activity, ABTS radical scavenging activity, •OH radical scavenging activity and FRAP, and the results were compared (Figure 2). Compared with the positive control, four EO samples did not show obvious reducing power to reduce the TPTZ-Fe (III) complex to the TPTZ-Fe (II) complex, which was similar to the results of total reducing power (Figure 2D). Of note, SDE-B and SD-B extracted from *M. officinalis* bark showed a better total reducing power than that of the flower (SDE-F and SD-F) (Figure 2E). Furthermore, the activity of these samples on •OH scavenging followed the following order: SDE-F > SD-B > SD-F > SDE-B (Figure 2C). At the maximum concentration, the •OH values of SDE-F, SD-B, SD-F and SDE-B were 36.43%, 28.79%, 21.64% and 15.53%, respectively, which is much lower than that of vit C (92.65%) at the same concentration point.

As is shown in Figure 2A, the DPPH• radical scavenging capacity of all EO samples at concentrations of 6.25–200 μg/mL gradually enhanced in a concentration-dependent manner. The DPPH• scavenging values of SDE-B, SD-B, SD-F and SDE-F were 55.04%, 54.34%, 54.21% and 53.86% at 200 μg/mL, respectively, which exceeded that of vit C at 50 μg/mL (43.02%). Figure 2B displayed that EOs from *M. officinalis* exhibited ABTS•+ free radical scavenging activity but no dose-dependent manner. At concentrations of 6.25–200 μg/mL, the ABTS•+ scavenging activity of SDE-B, SDE-F and SD-B increased from 22.32% to 31.34%, which were all higher than that of vit C at 100 μg/mL (19.98%). Unexpectedly, SD-F exhibited the lowest ability to quench ABTS•+. In conclusion, the total reducing power of EOs from *M. officinalis* was low, which was also similar for the hydroxyl free radical scavenging ability and ferric reducing antioxidant power. However, EOs from *M. officinalis* had a certain ability to scavenge DPPH and ABTS•+ free radicals. In comparison, Eos from *M. officinalis* bark (SDE-B and SD-B) exhibited a stronger anti-oxidant effect than that of the flower in vitro. It was also possible that the stronger anti-oxidant activities of SDE-B and SD-B were a result of the samples being rich in Eudesmol, which might produce anti-oxidant effects.

### 3.3. Anti-Tumor Activity

Recently, more and more research has shown that EOs also inhibited the proliferation of tumor cells [32,33]. EOs extracted from *Lippia citriodora murine* could significantly inhibited the growth of DA3 breast cancer cells in vitro and induce apoptosis; meanwhile, they inhibited the size of developing tumors in the DA3 murine tumor model [34]. In our study, the anti-tumor activity of EOs from *M. officinalis* were evaluated using three tumor cell lines including the human hepatocellular HepG2 cell line, breast carcinoma cancer MDA-MB-231 cell line and human lung carcinoma H1299 cell line. Cytotoxicity of EOs was determined using the human liver L02 cell line. Cells of L02, HepG2, H1299, MDA-MB-231 and RAW264.7 are preserved in our laboratory. 

**Figure 3:**
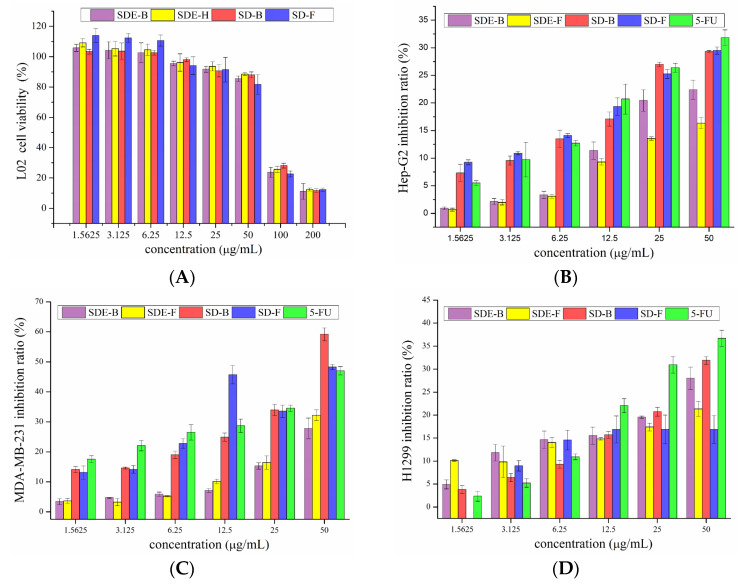
Cytotoxicity of SDE-F, SDE-B, SD-F and SD-B toward (**A**) L02 and inhibitory effects on Hep-G2 cells (**B**), MDA-MB-231 cells (**C**) and H1299 cells (**D**).

The survival rate of L02 cells with the addition of EO samples is shown in Figure 3A. The survival rates of L02 cells upon treatment with four EO samples were high (>80%) at low and medium concentrations (12.5, 25, 50 μg/mL), and low (<30%) at high concentrations (100, 200 μg/mL). Therefore, concentrations of EOs from *M. officinalis* ranging from 1.5625 to 50 μg/mL were employed for subsequent tests.

As is shown in Figure 3B, four EO samples exhibited significant inhibitory effects on Hep-G2 cells in a dose-dependent manner. Remarkably, the inhibitory rates of EOs extracted by the SD method were higher than that of 5-Fu at low concentrations (1.5625, 3.125, 6.25 μg/mL). With the increase in concentration, the inhibitory rates of SD-B and SD-F were significantly increased from 17.08 and 19.36 to 29.32 and 29.48% at the concentrations of 12.5–50 μg/mL, comparable to 5-Fu (20.71 to 31.84%). In the range of experimental concentration, EOs extracted by the SD method all exerted better anti-tumor effects than that of the SDE method. Previous reports [35,36,37] have shown that D-limonene can prevent and treat hepatocellular carcinoma, indicating that D-limonene, which represented 31.17, 8.92, 25.44 and 21.35% of SDE-F, SDE-B, SD-F and SD-B, respectively, might be responsible for the potent anti-proliferation activity on HepG2 cells of EOs from *M. officinalis*.

The inhibitory effects of EOs from *M. officinalis* on MDA-MB-231 cells are shown in Figure 3C. Different EO samples exhibited different inhibitory effects on MDA-MB-231 cells. SDE-B and SDE-F demonstrated moderate anti-cancer activities with the highest inhibitory rates at 27.80 and 32.23%, respectively, at 50 μg/mL. Specifically, SD-B and SD-F exerted excellent anti-cancer activities, where both inhibitory rates (59.21 and 48.27%, respectively) exceeded that of the positive control, 5-Fu (47.04%) at 50 μg/mL. Consistent with the cytotoxic activity on HepG2 cells, SD-B and SD-F extracted by the SD method showed higher inhibitory rates on MDA-MB-231 cells than SDE-extracted EOs.

Compared with the anti-cancer effects on HepG2 and MDA-MB-231 cells, EOs from *M. officinalis* showed a lower inhibitory effect on H1299 cells (Figure 3D). SD-B displayed the greatest inhibitory effects on H1299 cells at high concentrations of 25 and 50 μg/mL, followed by SDE-B, SDE-F and SD-F. Specifically, SD-B and SDE-B extracted from *M. officinalis* bark had stronger inhibitory effects on H1299 cells.

### 3.4. Anti-Inflammatory Activity

#### 3.4.1. Effect of EOs on LPS-Induced NO and iNOS in RAW 264.7 Cells

Many pro-inflammatory mediators, including NO and pro-inflammatory cytokines such as IL-1β, IL-6 and TNF-α, were involved in the regulation of the inflammatory process, and their overproduction and secretion might lead to various diseases including atherosclerosis, rheumatoid arthritis and even cancer [38,39,40]. LPS-induced RAW 264.7 cells, representing one common in vitro anti-inflammatory model [41], were used to evaluate the inflammatory activity of EOs from *M. officinalis*. Initially, MTT assays were carried out to evaluate the cytotoxicity of EO samples on RAW 264.7 cells. The survival rate of RAW 264.7 cells was less than 20% at a concentration of 100 μg/mL and more than 80% at concentrations of 12.5, 25 and 50 μg/mL. Therefore, 50 μg/mL was selected as the max concentration for subsequent experiments.

**Figure 4:**
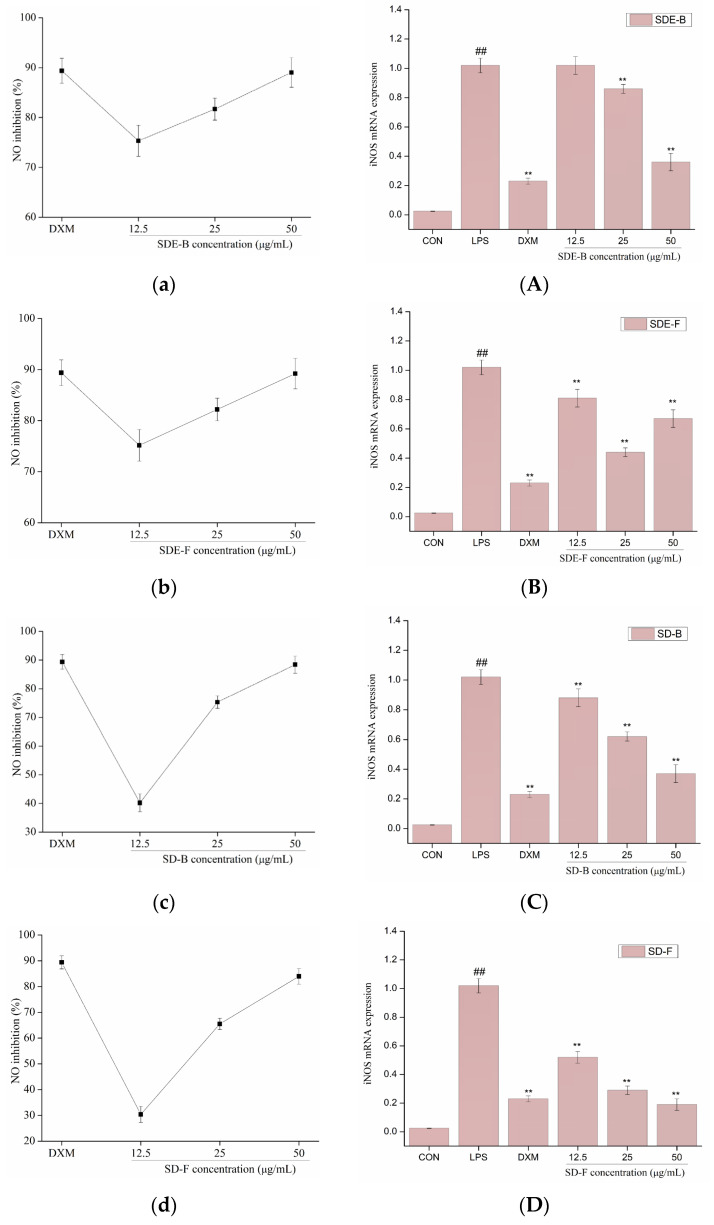
Effects of SDE-B, SDE-F, SD-B and SD-F on NO production (**a**–**d**), and iNOS mRNA expression (**A**–**D**) on LPS-induced RAW 264.7 cells. All experiments were run in triplicate, and data showed the mean ± SD values. (**) *p* < 0.01 compared to the LPS-treated group, while (##) *p* < 0.01 compared to the control group.

NO production was closely related to gene expression levels of iNOS. Activation of iNOS could produce a lot of NO by acting on the amino acid L-arginine. The production of NO and the mRNA expression of iNOS were determined in LPS-induced RAW264.7 cells with the addition of EOs from *M. officinalis*, and the results are shown in Figure 4. LPS could significantly induced the expression of iNOS at the level of transcription in RAW 264.7 cells, and result in the subsequent overproduction of NO, while four EOs isolated from *M. officinalis* remarkedly converted these events in a dose-dependent manner. Of note, all EO samples at a concentration of 50 μg/mL significantly inhibited NO production, comparable to the positive control DXM. Consistent with the downregulation of NO production, four EOs (especially SD-B and SD-F) also suppressed the mRNA expression of iNOS at the max concentration, and exhibited a closer anti-inflammatory effect to DXM. The results indicated that the EOs extracted from *M. officinalis* inhibited NO production via downregulation of the iNOS expression at the mRNA level in RAW 264.7 cells. An increasing number of previous studies also provided evidence for this. For example, Chu et al. reported that the essential oil from *Cinnamomum camphora* (Linn.) Presl leaves inhibits LPS-induced NO production through suppression of iNOS mRNA expression [42].

#### 3.4.2. Effect of EOs on LPS-Induced IL-6 and TNF-α in RAW 264.7 Cells

**Figure 5:**
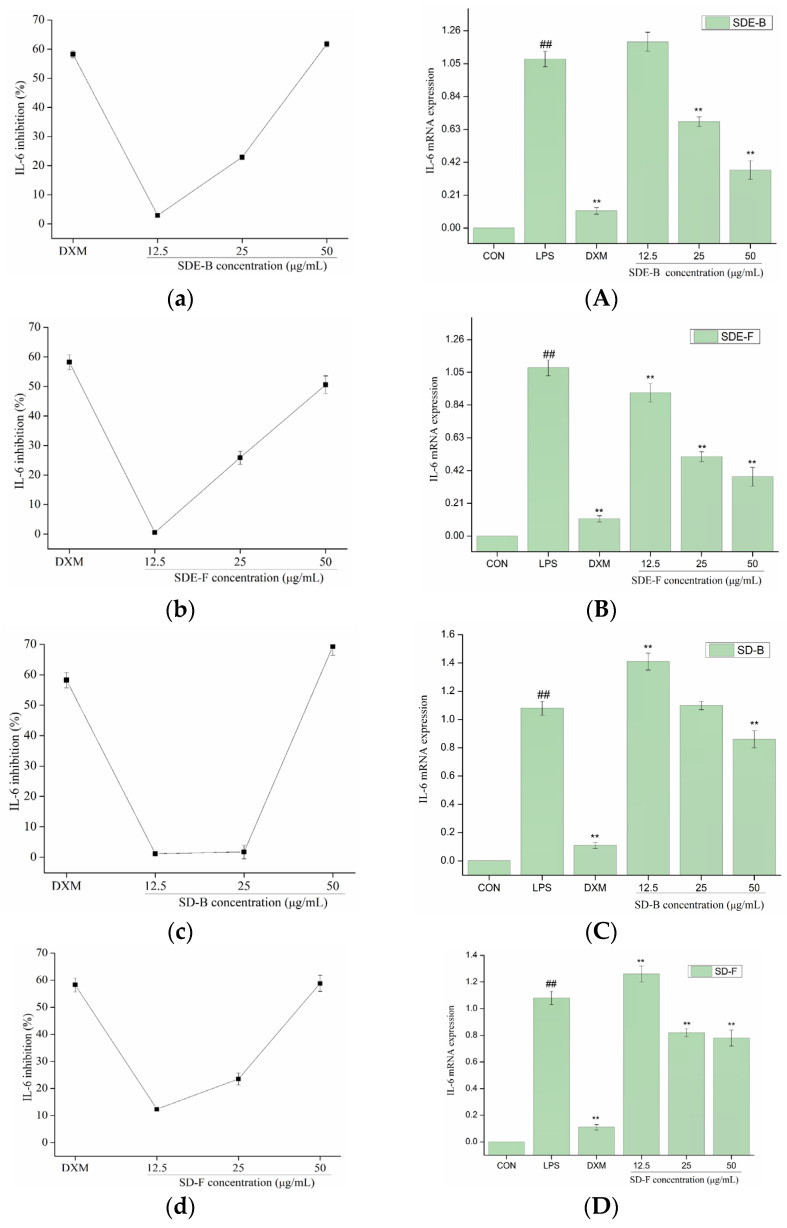
Effects of SDE-B, SDE-F, SD-B and SD-F on IL-6 production (**a**–**d**), and IL-6 mRNA expression (**A**–**D**) on LPS-induced RAW 264.7 cells. All experiments were run in triplicate, and data showed the mean ± SD values. (**) *p* < 0.01 compared to the LPS-treated group, while (##) *p* < 0.01 compared to the control group.

**Figure 6:**
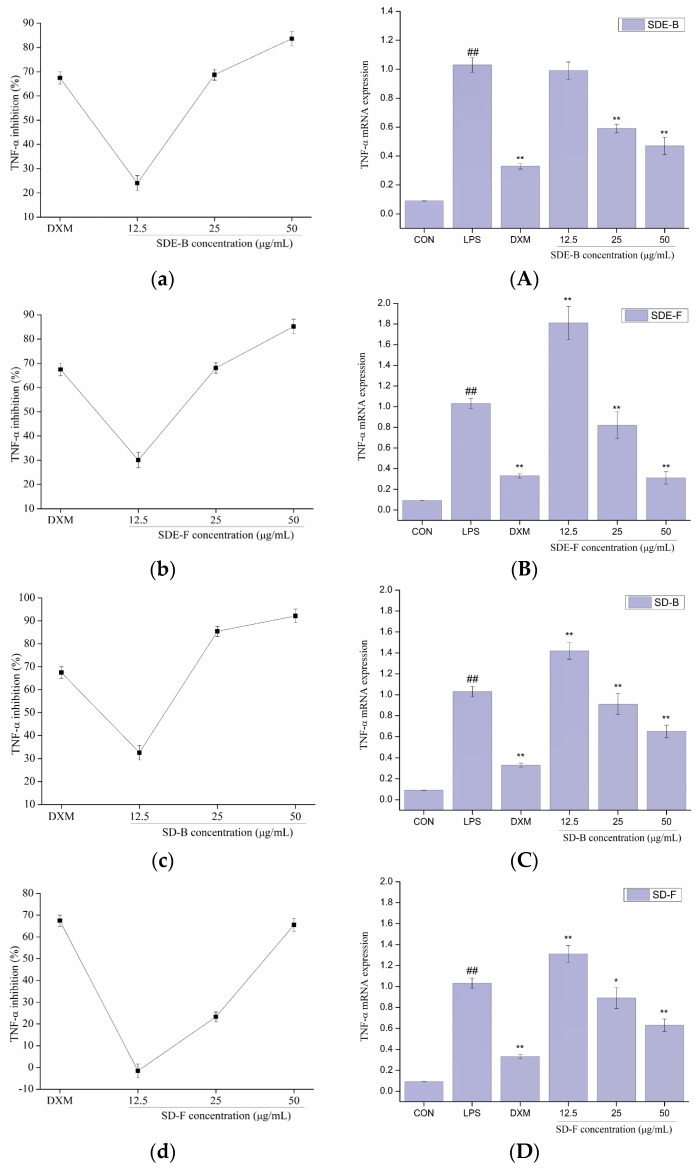
Effects of SDE-B, SDE-F, SD-B and SD-F on TNF−α production (**a**–**d**), and TNF−α mRNA expression (**A**–**D**) on LPS−induced RAW 264.7 cells. All experiments were run in triplicate, and data showed the mean ± SD values. (*) *p* < 0.05 and (**) *p* < 0.01 compared to the LPS-treated group, while (##) *p* < 0.01 compared to the control group.

In order to further evaluate the anti-inflammatory effect of EOs from *M. officinalis*, the secretion of pro-inflammatory cytokines including IL-6 and TNF-α were investigated. As shown in Figure 5 and Figure 6, four EOs significantly suppressed the secretion of IL-6 and TNF-α in RAW 264.7 cells, which were stimulated by LPS. After treatment with SDE-B, SDE-F and SD-F, IL-6 secretion decreased in a concentration-dependent manner; the inhibitory ratio at the concentration of 50 μg/mL was comparable to that of the positive control DXM. According to Figure 5c, SD-B only showed a strong inhibitory effect on the secretion of IL-6 at the max concentration. Additionally, all EO samples could remarkedly inhibit TNF-α release and decreased in the order of SD-B (92.17%), SDE-F (85.19%), SDE-B (83.64%) and SD-F (65.50%) at a concentration of 50 μg/mL (Figure 6a–d). Specifically, the inhibitory ratios of SD-B, SDE-F and SD-F exceeded that of DXM (67.44%) at a moderate concentration, while SD-F showed a slightly lower inhibitory effect on TNF-α secretion than the other three EOs.

The mRNA expressions of IL-6 and TNF-α were also determined using RT-PCR. Figure 5 and Figure 6 show that LPS treatment led to a significant increase in IL-6 and TNF-α mRNA expression compared to the control group. However, this increase was dramatically inhibited when EOs at a high concentration were administrated. Consistent with the data in Figure 5A–D, SDE-F and SDE-B showed stronger suppression effects on the expression of IL-6 at the mRNA level than SD-F and SD-B at low and moderate concentrations. Simultaneously, four EOs displayed moderate down-regulation effects at a moderate concentration and no effects at a low concentration on the mRNA level of TNF-α. According to Figure 6A–D, EOs from *M. officinalis* showed a higher inhibitory effect on TNF-α production than that on TNF-α gene expression. One possible explanation would be that TNF-α acted through two transmembrane receptors including TNFR1 and TNFR2 [43]. Another reason could be that the production of some other pro-inflammatory mediators, such as IL-6 and IL-1ß, could also regulate the secretion of TNF-α from activated macrophages [44].

## 4. Conclusions

The yields of four EOs extracted from the *M. officinalis* flower and bark by the SDE and SD methods were 0.121 ± 0.0012% (SDE-F), 0.223 ± 0.0017% (SDE-B), 0.110 ± 0.0015% (SD-F) and 0.218 ± 0.0012% (SD-B), respectively. D-limonene and Eudesmol were the main components of the Eos of the flower and bark. The component numbers of SDE-F (29) and SDE-B (26) were also much higher than that of SD-F (24) and SD-B (19). GC–MS results indicated that the SDE method could obtain higher yields of EOs and more components than the SD method. SD-F and SD-B exerted better anti-tumor effects than SDE on HepG2 and MDA-MB-231 cells. SD-B and SD-F showed high inhibitory rates on MDA-MB-231 cells (59.21 and 48.27%), even exceeding that of the positive control, 5-Fu (47.04%) at 50 μg/mL. Four EOs at high concentrations exhibited excellent anti-inflammatory activities through regulation of NO production and pro-inflammatory cytokine secretion in LPS-induced RAW 264.7 cells. The EOs from *M. officinalis* also remarkedly suppressed the mRNA expression of iNOS, IL-6 and TNF-α at max concentrations. In conclusion, the essential oils of *M. officinalis* isolated by different methods in this study showed excellent anti-tumor and anti-inflammatory activities, which lays the foundation for the development and utilization of *M. officinalis*. The future focus will be on the in-depth investigation of the anti-tumor and anti-inflammatory mechanisms and might be beneficial to the application of *M. officinalis* in food and drug enterprises.

## Figures and Tables

**Table 1:** Chemical composition of essential oils extracted from *Magnolia officinalis* Rehd. et Wils. identified by GC/MS.

Retention Time	Compound	CAS nr	Molecular Formula	Molecular Weight	Relative Percent Content/%			
					SDE-F	SDE-B	SD-F	SD-B
3.943	9,12-Octadecadienoyl chloride, (Z,Z)-	1509-85-9	C_18_H_31_O_2_	279.4375	- ^a^	0.28	0.84	0.13
3.122	Z-(13,14-Epoxy) tetradec-11-en-1-ol acetate	-	C_16_H_28_O_3_	268.3917	-	7.37	5.49	12.35
4.842	1R-α-Pinene	7785-70-8	C_10_H_16_	136.2340	2.34	0.69	-	-
5.072	Camphene	79-92-5	C_10_H_16_	136.2340	-	0.66	-	0.78
5.488	Sabinene	3387-41-5	C_10_H_16_	136.2340	7.91	-	-	-
5.663	β-Pinene	18172-67-3	C_10_H_16_	136.2340	12.25	-	4.26	-
6.084	1,8-Nonadiene-3-ol	159010-02-3	C_9_H_16_O	140.2227	0.47	-	-	-
6.2	o-Cymene	527-84-4	C_10_H_14_	134.2182	4.70	-	-	-
6.201	β-Cymene	535-77-3	C_10_H_14_	134.2182	-	3.02	-	-
6.201	trans-Verbenyl caprate	-	C_20_H_34_O_2_	306.4828	-	-	-	0.29
6.263	D-Limonene	5989-27-5	C_10_H_16_	136.2340	31.17	11.37	25.44	21.35
6.318	(Z,Z,Z)-3,6,9-Nonadecatriene	89353-62-8	C_19_H_34_	262.4733	-	0.67	-	-
6.521	β-cis-Ocimene	3338-55-4	C_10_H_16_	136.2340	0.65	-	-	-
6.63	9-Octadecenoic Acid (Z)-, 2-hydroxy-1-(hydroxyMethyl) ethyl ester2-	123-94-4	C_21_H_40_O_4_	356.5399	-	0.56	1.01	0.60
6.713	3-Carene	13466-78-9	C_10_H_16_	136.2340	0.91	-	-	-
7.171	cis-Sabinene hydrate	15537-55-0	C_10_H_18_O	154.2493	2.16	-	-	-
7.313	Linalool	78-70-6	C_10_H_18_O	154.2493	5.45		3.62	-
7.338	Perillene	539-52-6	C_10_H_14_O	150.2176	8.32			
8.23	Hexadecanoic acid, (2-phenyl-1,3-dioxolan-4-yl) methyl ester, cis-	42495-31-8	C_26_H_42_O_4_	418.61				0.15
8.521	Terpinen-4-ol	562-74-3	C_10_H_18_O	154.2493	0.91	-	-	-
8.65	Thymol	89-83-8	C_10_H_14_O	150.2176	0.49	-	-	-
8.683	8-diene-2-ol	22626-43-3	C_10_H_16_O	152.2334	1.09	-	-	-
8.729	α-Terpineol	98-55-5	C_10_H_18_O	154.2493	2.08	-	1.80	-
8.834	cis-Vaccenic acid	593-39-5	C_18_H_34_O_2_	282.4614	-	-	0.27	-
8.837	(-)-Myrtenol	19894-97-4	C_10_H_16_O	152.2334	1.68	-	1.15	-
9.591	Myrtenol	515-00-4	C_10_H_16_O	152.2334	0.62	-	-	-
9.601	Z,E-3,13-Octadecadien-1-ol	-	C_18_H_36_O	268.4778	-	-	-	0.13
10.064	cis-Vaccenic acid	506-17-2	C_18_H_34_O_2_	282.4614	-	0.09	0.41	0.15
10.066	Octadecanal, 2-bromo-	56599-95-2	C_18_H_35_BrO	347.37	-	-	-	0.23
10.133	cis-Carveol	1197-06-4	C_10_H_16_O	152.2334	0.35	-	-	-
10.293	Bornyl acetate	76-49-3	C_12_H_20_O_2_	196.286	0.61	1.51	-	-
10.954	γ-Muurolene	30021-74-0	C_15_H_24_	204.3511	1.20	-	10.58	-
11.87	α-Copaene	3856-25-5	C_15_H_24_	204.3511	0.48	-	-	-
11.956	2H-Pyran, 2-(7-heptadecynyloxy) tetrahydro-	56599-50-9	C_22_H_40_O_2_	336.5518	-	0.09	-	-
12.238	E-2-Methyl-3-tetradecen-1-ol acetate	-	C_17_H_32_O_2_	268.4347	-	-	-	0.11
12.468	Undec-10-ynoic acid, octadecyl ester	-	C_29_H_54_O_2_	434.7378	-	0.13	-	-
12.827	Formic acid, 3,7,11-trimethyl-1,6,10-dodecatrien-3-yl ester	-	C_16_H_26_O_2_	250.3764	-	0.12	-	0.09
13.364	Nerylacetone	3879-26-3	C_13_H_22_O	194.3132	-	-	1.18	-
13.42	β-Sesquisabinene	58319-04-3	C_15_H_24_	204.3511	1.50	-	-	-
13.918	(-)-Isolongifolol, methyl ether	-	C_16_H_28_O	236.3929	-	0.10	-	-
14.16	1-heptatriacotanol	105794-58-9	C_37_H_76_O	536.9987	-	0.58	-	-
14.618	β-Methylionone	127-43-5	C_14_H_22_O	206.3239	-	0.12	-	-
14.607	2,4-Di-tert-butylphenol	96-76-4	C_14_H_22_O	206.3239	0.32	-	0.34	-
14.456	α-Agarofuran	5956-12-7	C_15_H_24_O	220.3505	-	1.49	-	-
14.718	Ethyl iso-allocholate	47676-48-2	C_26_H_44_O_5_	436.6245	-	-	0.40	-
14.857	(+)-δ-Cadinene	483-76-1	C_15_H_24_	204.3511	1.55	-	12.17	-
15.051	Phenol,2-(1,1-dimethylethyl)-4-(1-methyl-1-phenylethyl)-	56187-92-9	C_19_H_24_O	268.39	-	0.08	1.27	0.41
15.299	α-Calacorene	21391-99-1	C_15_H_20_	200.3193	0.32	-	-	-
15.293	2-([(Dimethylamino)methylene] amino)-3-(3-chloro-4-ethyloxy-phenyl) propanoic acid, ethyl ester	-	C_16_H_23_ClN_2_O_3_	326.8184	-	0.12	-	-
15.501	5,6,7,8,9,10-Hexahydro-9-methyl-spiro[2H-1,3-benzoxazine-4,1′-cyclohexane]-2-thione	-	C_14_H_24_NOS	254.4114	-	0.36	-	0.21
15.615	Nerolidol	7212-44-4	C_15_H_26_O	222.3663	3.98	-	10.79	-
16.14	Caryophyllene oxide	1139-30-6	C_15_H_24_O	220.3505	0.91	2.28	1.15	0.77
16.143	Diepicedrene-1-oxide	-	C_15_H_24_O	220.3505	-	-	1.27	-
16.681	Humulene epoxide 2	19888-34-7	C_15_H_24_O	220.3505	-	0.87	-	-
17.272	γ-Eudesmol	1209-71-8	C_15_H_26_O	222.3663	-	12.39	-	12.90
17.522	β-Eudesmol	473-15-4	C_15_H_26_O	222.3663	3.11	12.55	0.46	8.10
17.576	α-Eudesmol	473-16-5	C_15_H_26_O	222.3663	1.12	40.17	0.96	40.36
17.693	1,3-dioxane, 5-(hexadecyloxy)-2-pentadecyl-, trans-	34315-34-9	C_35_H_70_O_3_	538.9285	-	-	0.23	-
17.992	androsta-1,4,6-triene-3,17-dione	633-35-2	C_19_H_22_O_2_	282.381	-	-	-	0.31
17.997	Cadalene	483-78-3	C_15_H_18_	198.3034	-	0.32	-	-
19.272	Benzofuran-6-ol-3-one, 2-(4-ethoxycarbonyl) benzylidene-	-	C_18_H_14_O_5_	310.3008	-	-	0.59	-
20.305	trans-Geranylgeraniol	24034-73-9	C_20_H_34_O	290.4834	-	-	1.69	-
20.484	Farnesyl alcohol	4602-84-0	C_15_H_26_O	222.3663	-	-	31.89	-

^a^ “-” = Not found or does not exist.

## Data Availability

The original contributions presented in the study are included in the article, further inquiries can be directed to the corresponding author.

## Abbreviations

ABTS•+: 2,2′-azinobis (3-ethylbenzothiazoline-6-sulfonic acid) diammonium salt radical cation; DPPH•: 1,1-diphenyl-2-picrylhydrazyl radical; EOs: essential oils; FRAP: ferric reducing antioxidant power assay; GC–MS: Gas Chromatography–Mass Spectrometry; HepG2: human hepatocarcinoma cells; HRE: heating reflux extraction; iNOS: nitric oxide synthase; IL-1β: interleukin-1β; IL-6: interleukin-6; L02: human hepatocyte cells; LPS: lipopolysaccharide; MDA-MB-231: human breast cancer cells; NO: nitric oxide; •OH: hydroxyl radical; SD: steam distillation; SD-B: EO extracted by the SD method from bark; SD-F: EO extracted by the SD method from the flower; SDE: simultaneous distillation extraction; SDE-B: EO extracted by the SDE method from bark; SDE-F: EO extracted by the SDE method from the flower; SFE: supercritical fluid extraction; TNF-α: tumor necrosis factor-α; TPTZ: 1,3,5- tri(2-pyridyl)-2,4,6-triazine.
